# DNA Sequence Analysis of South African *Helicobacter pylori* Vacuolating Cytotoxin Gene *(vacA)*

**DOI:** 10.3390/ijms12117459

**Published:** 2011-10-31

**Authors:** Nicoline F. Tanih, Lucy M. Ndip, Roland N. Ndip

**Affiliations:** 1 Microbial Pathogenicity and Molecular Epidemiology Research Group, Department of Biochemistry and Microbiology, Faculty of Science and Agriculture, University of Fort Hare, P/Bag X1314, Alice 5700, South Africa; E-Mail: rndip@ufh.ac.za; 2 Department of Biochemistry and Microbiology, Faculty of Science, University of Buea, Box 63, Buea, Cameroon; E-Mail: lndip@yahoo.com

**Keywords:** diversity, *Helicobacter pylori*, vacuolating cytotoxin gene (*vacA*), South Africa

## Abstract

Sequence diversity and population structures can vary widely among pathogenic bacteria species. In some species, all isolates are highly similar, whereas in others most of the isolates are distinguished easily. *H. pylori* is known for its wide genetic diversity amongst the various strains most especially in the genes involved in virulence. The aim of this study was to evaluate by PCR and sequence analysis, the genetic profile of *H. pylori vacA* gene *(*s1, s2, m1 and m2). We sequenced small DNA segments from 13 *vacA*s1, 10 *vacA*m2, 6 *vacA*m1 and 6 *vacA*s2 strains which were amplified with amplicon size of 259/286 bp, 290 bp and 352 bp for *vacA*s1/s2, m1 and m2 respectively. Based on similarities among our strains accession numbers were provided for seven *vacA*s1 (HQ709109–HQ709115), six *vacA*s2 (JN848463–JN848468), six *vacA*m1 (JN848469–JN848474) and six *vacA*m2 (HQ650801–HQ650806) strains. Amongst the strains studied, 98.07%, 98.58%, 97.38% and 95.41% of *vacA*s1, *vacA*s2, *vacA*m1 and *vacA*m2 of the strains were conserved respectively. Findings of this study underscores the importance of understanding the virulence composition and diversity of *H. pylori* in South Africa for enhanced clinico-epidemiological monitoring and pathophysiology of disease.

## 1. Introduction

*H. pylori* is a gastric pathogen that infects more than 50% of the world’s population; is the major cause of a number of gastro duodenal pathologies in infected patients [1,2], however, only a small subset of infected people experience *H. pylori*-associated illnesses such as chronic gastritis, peptic ulcer disease, gastric carcinoma and mucosa-associated lymphoid tissue (MALT) lymphoma [3,4]. Strains of *H. pylori* are renowned for tremendous variation in their genes [5–7]; in some species, all isolates are highly similar, whereas in other species, any two unrelated isolates can be easily distinguished from most others by DNA fingerprinting or sequencing of the representative gene segments [5,8].

*H. pylori* is frequently considered panmictic in population structure in that genetic recombination is so frequent in its DNA sequences and generates linkage equilibrium [9]. Typically some 3 to 5% DNA sequence divergence exist between isolates even in essential genes [7]. This diversity probably reflects a combination of factors including: mutation, recombination among divergent lineages, gene transfer from unrelated species and preferential transmission among family members and people in close contact which is propagated as a result of lack of selection for just one or a few genotype that might be best adapted for all humans [6,10]. Also, the knowledge that humans differ in traits that could be important to individual strains such as specificity, strength of the immune system, inflammatory responses and availability and distribution of adhesion molecules for the organism would select for divergence among *H. pylori* strains [7].

Clinically, the outcome of infection is variable and is considered to relate to bacterial virulence factors which may vary with sequence type, host and the environment [6,11]. *VacA* is secreted as vacuolating cytotoxin thought to be involved in ulcerogenesis whose sequence variability seems to reflect mosaicism (*vacA*s and m) [6]. Direct sequencing, multilocus sequence typing (MLST) and other high throughput typing schemes has indicated that, different *H. pylori* genotypes predominate in different parts of the world and human ethnic groups [6,8]. In particular, African strains seem to be distinct from those of other continents [6,12]. Mukhopadhyay *et al.* [8] reported tremendous diversities amongst strains of India, ethnic European and East Asia in their study. Also, DNA sequence motifs predominating in two virulence-associated genes *vacA* (vacuolating cytotoxin) and *cagA* (cytotoxin associated gene), in strains from the United States and Europe were found to differ from those predominating in southern coastal China and Japan [7].

Colonization with *H. pylori* is very common in South Africa, as in other developing countries [13–16]. Most studies of *H. pylori* have concentrated on virulence factors determination rather than sequence analysis of these factors. This is because a good knowledge of the burden of a particular genotype will enhance better understanding of its molecular epidemiology and foster drug design in that regard. Limitations in resources also make it difficult to embark on highly informative technique like sequencing. However, sequence typing provides intricate information on the nucleotide diversity which cannot be accomplished by polymerase chain reaction alone (virulence factors determination).

Recently, we genotyped strains of *H. pylori* in the Eastern Cape Province of South Africa and found different combinations of genotypes with s1 and m2 being the most prevalent genotype combination [17]. However, there is a dearth of information on comparative sequence analysis of *H. pylori* strains in this part of the world. Suerbaum *et al*. [18] analyzed for diversity and linkage equilibrium between South Africa, Canada and Germany. Their study was based on *H. pylori* strains isolated from colored patients with gastroduodenal disease in Cape Town which is anthropologically distinct (from native South African populations) with ancestry from Western Europeans, South East Asians and South Africans (Hottentots). Also, Kersulyte *et al*. [7] studied South African strains from native black Africans that are residents of Soweto. In the current study we compared sequences of *vacA* (*vacA*s1, s2, m1 and m2 alleles) derived from strains of *H. pylori* in different racial groups (white, colored, blacks) in the Eastern Cape province of South Africa with one another and emphatically we further compared *vacA*s1 and m2 from our study with those from other regions because the combination s1m2 was the most prevalent in this study. Our data provide overwhelming evidence of diversity in *H. pylori* strains.

## 2. Results and Discussion

### 2.1. Results

Agarose gel electrophoresis of the samples that were detected by PCR revealed a single band corresponding to the expected amplicon size 259/286 bp, 290 bp and 352 bp for *vacA*s1/s2, m1 and m2 respectively as previously detailed (17).

#### Genetic Diversity of *vacA*

We compared DNA sequences through blasting and alignments of *vacA*s1, s2, m1 and m2 nucleotides found in *H. pylori* strains in our study area with those already deposited in the gene bank from different study areas. Sequenced amplicons for *vacA*s1 was 92–98% identical to each other and to those submitted already in the genebank while for *vacA*s2, *vacA*m1 and *vacA*m2, it was 90–98%, 87–99% and 89–98% respectively. Six of our *vacA*s1 sequences were homologous to one or more of the thirteen sequences in our collection for *vacA*s1 while four of *vacA*m2 sequences also presented with homology amongst our sequences. All six strains sequenced for both *vacA*s2 and *vacA*m1 were different from each other. In this regard, we submitted seven *vacA*s1 [3 colored (*vacA*s1 SA1, *vacA*s1 SA4, *vacA*s1 SA7*)*, 3 blacks (*vacA*s1 SA2, *vacA*s1 SA3, *vacA*s1 SA6*)* and 1 white patient (*vacA*s1 SA5)] and six *vacA*m2 [3 colored (*vacA*m2 SA1, *vacA*m2 SA4, *vacA*m2 SA5) and 3 black patients (*vacA*m2 SA2, *vacA*m2 SA3, *vacA*m2 SA6), six *vacA*s2 (4 blacks and 2 colored), and six *vacA*m1 (3 blacks, 2 colored and 1 white)] sequences in the genebank. The various strains were coded as SA = South Africa and numerical to denote strain number. *H. pylori vacA*s1 obtained in our study were deposited in the GenBank and assigned accession numbers (HQ709109–HQ709115) while those for *vacA*m2, *vacA*s2 and *vacA*m1 were (HQ650801–HQ650806), (JN848463-JN848468) and (JN848469–JN848474) respectively. Nucleotides for the sequenced genes in this study were conserved except for the mutations (missense and silence) which we observed.

**Figure 1:**
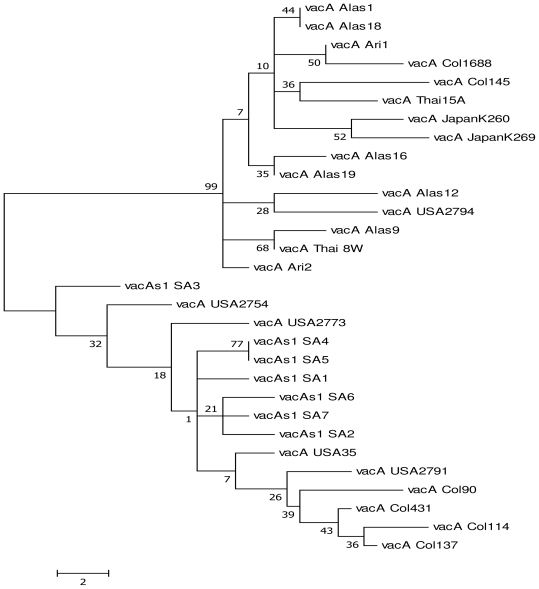
Maximum parsimony analysis of *vacA*s1 sequences from clinical isolates showing that phylogenetic analysis between strains is not distinct. There is clustering between the strains. Branches with significant bootstrap support are indicated; origins of *H. pylori* strains are labelled with a code: SA—South Africa, USA—USA, Col—Colombia, Thai—Thailand, Alas—Alaska, Ari—Arizona, Japan-Japan; Bar indicates 2 nucleotide substitution per site. Our *vacA*s1 sequences were compared with (AB057218.1, AB057223.1, AB057222.1, AB057221.1 AB057219.1, AB057214.1, AB057216.1, AB057217.1, AB057211.1, AB057215.1, AB057190.1, AB057188.1, AB057187.1, AB057185.1, AB057178.1, AB057175.1, AB057184.1, AB057182.1, AB057167.1, AB057164.1, AB057166.1, AB057140.1, AB057139.1).

**Figure 2:**
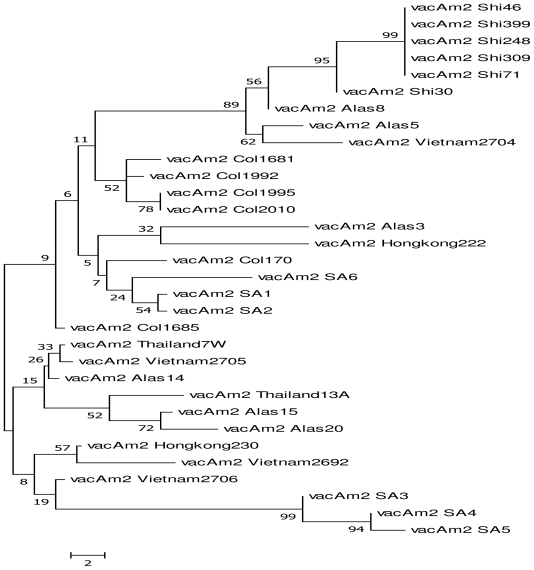
Maximum parsimony analysis of *vacA*m2 sequences from clinical isolates showing that phylogenetic analysis between strains is not distinct. There is clustering between the strains. Branches with significant bootstrap Support are indicated; origins of *H. pylori* strains are labelled with a code: SA—South Africa, USA—USA, Col—Colombia, Thailand—Thailand, Vietnam—Vietnam, Alas—Alaska, and Hongkong—Hongkong, Shi—Shi; Bar indicates 2 nucleotide substitutions per site. *vacA*m2 sequences were compared with the following sequences with accession numbers (AB057325.1 AB057323.1, AB057328.1, AB057327.1, AB057326.1, AB057334.1, AB057301.1, AB057290.1, AB057295.1, AB057302.1, AB057292.1, AB057307.1, AB057277.1, AB057276.1, AB057284.1, AB057286.1, AB057280.1, AB057281.1, AB057279.1, AB057282.1, GU064493.1, GU064499.1, GU064498.1, GU064495.1, GU064496.1, GU064497.1) derived from the gene bank.

We compared our sequences with other partial coding sequences existing in the genebank for both *vacA*s1 and m2 (as the combination s1m2 was most prevalent in our study area ) from the following regions: South Africa, USA, Colombia, Thailand, Vietnam, Alaska, Arizona, Japan, Hongkong and Shi (Figures 1 and 2). Results from comparison with other sequences depict clustering of the sequences which generally depicts similarities between the strains employed though with some differences.

### 2.2. Discussion

*H. pylori* is a slow growing microaerophilic spiral bacterium of medical importance; determination of its complete genome sequence [1,2,19], has enhanced understanding of its pathogenecity [19]. Specific genes and their sequences have been shown to be involved in the pathogenicity of *H. pylori* leading to a specific disease condition. Hence understanding nucleotide or protein variation that exists within a gene in *H. pylori* will help accelerate understanding of possible disease outcome. A significant level of variation at the nucleotide level is seen across the genome providing an explanation why the nucleotide base typing techniques offer high discriminatory power among independent *H. pylori* isolates [6,20]. We used direct sequencing to generate sequences from small DNA segments of *vacA* (s1, s2, m1 and *vacA*m2) genes which are highly associated with disease in *H. pylori* infection [20]. These sequences were compared to one another and to other strains already deposited in the gene bank. Nucleotide sequence diversity of *H. pylori* exceeds that of many other bacterial species. The observed differences in sequence diversity between different bacterial species can readily be explained by differences of population structure and population size. Furthermore, almost every nucleotide sequence from unrelated isolates is unique, an unprecedented situation [6]. Nucleotide sequences generated from this study from *vacA*s1, s2, m1 and *vacA*m2 exhibited some degree of divergence of 1.93%, 2.62%, 1.42% and 4.59% respectively. Our results are similar to the report of other authors who indicated high genetic diversity in *H. pylori* strains which renders it impossible to isolate identical strains from different or the same individual [18,19,21].

Divergence in alleles could in theory reflect high mutation rates or frequent recombination or a combination of both [6]. We found mutations among the nucleotide sequences in this study. Missense and silent mutations were the most frequently observed mutations, and based on the unique differences observed for the various strains, different Gene bank accession numbers were assigned to *vacA*s1 (HQ709109-HQ709115), *vacA*s2 (JN848463-JN848468), *vacA*m1 (JN848469-JN848474) and *vacA*m2 (HQ650801–HQ650806). Almost all polymorphism were synonymous substitutions (which did not affect the amino acid sequence), insertions and deletions. This data is in accord with the findings of Maiden *et al.* [22] who indicated that extensive DNA sequence data available for *H. pylori* genes show that transition mutations account for most of the inter-strain micro diversity. Also, mutation is considered as the key for phenotypic variation as well as ability of cellular adaptations to stress [9]. Great similarity was observed between our *vacA*s1 strains when compared to the standard strain U07145; also nucleotides of *vacA*s1 strains in our study area were 98.07% identical to each other while 1.93% were different. Also, high level of conservation was reported of the nucleotides in *vacA*m2 (95.41%) strains as opposed to 4.59% dissimilarity. Furthermore, we had 98.58% identity for *vacA*m1 and 97.38% identity for *vacA*s1 amongst our isolates while 2.62% and 1.42% difference was observed respectively. This finding is in line with that of Alm and Trust [20] who reported that significant variation is known to exist in *vacA H. pylori* sequence to an extend that every isolate seem to poses a unique sequence. However, we found six and four *vacA*s1 and *vacA*m2 sequences respectively to be identical to one or more of the sequences of the strains we studied. Our results accords the findings of Suerbaum and Achtman, [6] who found Cape Colored isolates to possess identical *flaB* and *vacA* sequences in their studies amongst South African strains. Meanwhile, it contradicts those of other investigators who found all strains from Canada and Germany to be unique, suggesting that the pool of different alleles may be smaller in some geographic regions than in Canada and Europe [6].

Our dissimilarity in results ties with the findings of Mukhopadhyay *et al.* [8] and Cover *et al*. [23] that reported high divergence of *vacA*m2 strains and suggested that there may be considerable sequence diversity among strains in the middle region of the *vacA* gene.. Also, evolutionary consideration and high rates of transmission favor the emergence of more virulent strains of the organism [24]. The differences observed are thought to have resulted due to selection pressures in different ancestral animal host rather than in humans [8]. More to that, Wirth *et al.* [24] reported wide genetic diversity in *H. pylori* strains from different region and even between ethnic groups in the same geographical region.

Sequence analysis has shown that different genotypes of *H. pylori* prevail in different geographical locations [8]. We compared the sequences derived from this study (s1m2) with those already deposited in the genebank using phylogenetic analysis by maximum parsimony (MP) (Figure 1 and 2). Our results reveal clustering of the strains employed which generally depicts close similarity between the strains though different. Figure 1 shows clustering of strains of *vacA*s1: SA1, SA2, SA4, SA5 and SA6 except for *vacA* SA3 which is far apart. The same applies to *vacA*m2 strains where SA1, SA2 and SA6 cluster around each other while SA3, SA4 and SA5 cluster. This clustering is irrespective of racial backgrounds be it colored, black or white. This is in line with the findings of Suerbaum and Achtman [6] who showed that 20 strains of *H. pylori* from 12 countries differed according to their sources. They added that six isolates from East Asians differed from the remainder of the world and were called the “Asian” clone. Three isolates from two Africans and one African American formed a second distinct group “Clone 2” and the other eleven isolates from Europe, America and Australia formed a heterogeneous third group. These results tie with the fact that isolates from a single individual, family member and a particular geographical region are frequently clonal; however the overall population structure of *H. pylori* is panmictic [25]. Comparison of our sequences (*vacA*) with the three African strains which have been completely sequenced and deposited in the gene bank [(J99 from Caucasian in Pulaski TN, but widely regarded as of the African strain type GenBank: AE001439.1; strain 908 (from West African immigrant in Bordeaux, France GenBank: CP002184.1 and Gambia94/24 GenBank: CP002332.1)] was impracticable because there were several protein identities for *vacA* in these whole genomes. This study however has as short coming small sample size. Large sample screening would have provided a better understanding of the various possible strains that would exist in this population.

## 3. Materials and Methods

### 3.1. Bacterial Strains

*H. pylori* strains employed in this study were isolated and identified from patients who underwent a complete physical examination, and history was taken by a resident gastro-enterologist. Endoscopic diagnoses were made by a single experienced endoscopist after informed consent and ethical clearance (protocol number EcDoH-Res 0002). Antral and corpus biopsy specimens each were obtained at endoscopy, and immediately placed in sterile bijou bottles containing 0.2 g/L of cysteine and 20% glycerol in brain-heart infusion (BHI) broth and transported in ice to the laboratory within 2 h of collection. Gastric biopsies were collected from patients and *H. pylori* was isolated on Columbia agar base supplemented with 7% sheep’s blood and Skirrow’s supplement containing trimethoprim (2.5 mg), vancomycin (5 mg) and cefsulodin(2.5 mg). Amphotericin (2.5 mg) was added to the medium. Recovered isolates were identified following standard microbiology and biochemical techniques [26]. A reference strain of *H*. pylori (NCTC 11638) was included as a positive control. Confirmed isolates were suspended in 20% glycerol and stored at −80 °C in a freezer (Sanyo, Japan) for future experiments.

### 3.2. Molecular Characterization

DNA extraction and PCR amplification were as reported in our previous study [17]. Briefly, DNA was extracted from a hundred strains. PCR analysis of the targeted genes was performed using Thermo-stat Taq DNA polymerase (ABgene, UK) and manufacturer-provided reaction buffer. The primers used to amplify the targeted genes were 5′-ATGGAAATACAACAAACACAC-3′ and 3′-CTGCTTGAATGCGCCAAAC-5′ for *vacA*s1/s2 while for *vacA*m1 and *vacA*m2 the primers were 5′-GTCAAAATGCGGTCATGG-3′ and 3′-CCATTGGTACCTGTAGAAAC-5′ and 5GGAGCCCCAGGAAACATTG-3′ and 3′- CATAACTAGCGCCTTGCAC-5′ respectively [2].

### 3.3. Sequence Analysis

We sequenced thirteen small DNA segment from *vacA*s1 (Seven from coloured patients, five from blacks and one white), ten *vacA*m2 strains (five colored and five blacks), six *vacA*s2 (4 blacks, 2 colored) and six *vacA*s1 (3 blacks, 2 colored and 1 white) (Iqaba, Pretoria, SA). Mutations and genetic diversity were analyzed by sequencing using a Big Dye Terminator DNA sequencing kit v3.1 (Applied Biosystems, UK) after purification of products using shrimp alkaline phosphatase. DNA sequence editing and analysis was performed with the programs Bioedit and EMBL- EBI- Clustalw2. Sequences were aligned using DNAMAN. Maximum parsimony, a non-parametric statistical method commonly used in computational phylogenetics for estimating phylogenies was used to generate trees for this study.

## 4. Conclusion

Our finding underscores the need of understanding the virulence composition and diversity of *H. pylori* in South Africa for enhanced clinico-epidemiological monitoring and pathophysiology of disease.
